# Critical care utilization associated with an electronic dance music festival in Las Vegas, Nevada: A retrospective cohort study of the electric daisy carnival

**DOI:** 10.1016/j.pmedr.2026.103439

**Published:** 2026-03-04

**Authors:** Jie Ren, Kavita Batra, Vidhani Goel, Taya Jensen, San Tran, Mutsumi Kioka

**Affiliations:** a Division of Pulmonary and Critical Care Medicine, Department of Internal Medicine, Kirk Kerkorian School of Medicine, University of Nevada, Las Vegas, Nevada, USA.; b Office of Research, Department of Medical Education, Kirk Kerkorian School of Medicine, University of Nevada, Las Vegas, Nevada, USA.; c School of Public Health, University of Nevada, Las Vegas, Nevada, USA.

**Keywords:** Electronic dance music festivals, Drug overdose, Critical care utilization, Harm reduction, Public health burden

## Abstract

**Introduction:**

Electronic dance music (EDM) festivals are associated with recreational drug use and medical emergencies, yet comparative data on the severity of festival-related overdoses are limited. This study compared hospital outcomes, particularly **critical care utilization,** between patients transported from the Electric Daisy Carnival (EDC) and those with non–festival-related overdoses during the same period.

**Methods:**

We conducted a retrospective cohort study of adults (≥18 years) presenting with drug or alcohol overdose to an urban tertiary care teaching hospital between May 17, 2019, and May 20, 2024. Patients were classified as EDC-related or non-EDC based on electronic medical record documentation. Group differences were assessed using chi-square and independent *t*-tests. Logistic regression evaluated predictors of intensive care unit (ICU) admission.

**Results:**

Of 1127 hospital admissions, 103 met eligibility criteria; 40 (38.80%) were EDC-related and 63 (61.20%) were non-EDC. EDC-related patients were younger (32.40 ± 8.50 vs. 44.30 ± 11.60 years, *p* < 0.001) but did not differ significantly from non-EDC patients in sex distribution, drug use patterns, Sequential Organ Failure Assessment (SOFA) scores, or QT intervals. ICU admission was higher among EDC-related patients compared with non-EDC patients (77.50% vs. 44.40%, *p* < 0.001). No significant differences were observed in total hospital length of stay, ICU length of stay, or mortality between groups. Logistic regression showed age and EDC status as the significant predictors of ICU admission.BMI demonstrated a borderline association whereas sex, QT interval, were not independently associated with ICU admission.

**Conclusion:**

EDC-related overdose patients had markedly higher likelihood of ICU admission compared with non-EDC overdose patients, despite similar clinical characteristics on presentation. The findings highlight the disproportionate resource burden associated with EDM festivals and underscore the need for enhanced harm-reduction strategies, improved on-site medical triage, and proactive planning by local hospitals and emergency services.

## Highlights

• Mass gatherings increase demand for critical care services.
• Festival-related overdose patients were admitted to intensive care more often.
• Clinical severity scores were similar between groups.
• Intensive care admission estimated resource utilization.
• Large music festivals strain local hospital capacity.

## 1 Introduction

Electronic dance music (EDM) festivals have become a prominent component of youth and young adult culture in the United States (US), drawing millions of attendees to hundreds of large-scale events each year (Apruzzese and Tompkins, 2023; JamBase, 2025). Although these festivals promote social connection and entertainment, they are consistently associated with a high prevalence of recreational drug use, particularly 3,4-Methylenedioxymethamphetamine (MDMA), amphetamines, cocaine, ketamine, and polysubstance combination substances (Alo and Kioka, 2024; Palamar et al., 2019). The physiological risks of these substances are amplified by environmental factors typical of festival settings including prolonged dancing, elevated ambient temperatures, dehydration, and overcrowding, which can precipitate hyperthermia, hyponatremia, acute organ dysfunction, and death (Palamar and Keyes, 2020). Recent epidemiologic studies indicate that drug use among EDM attendees continues to rise, with increasing prevalence of MDMA, cocaine, ketamine, and novel psychoactive substances, including synthetic cathinones (“Bath Salts”) and fentanyl analogs (Palamar et al., 2019; Palamar and Keyes, 2020). These adulterants are often undetected by users and contribute to unpredictable toxicity profiles, escalating the risk of severe overdose requiring advanced medical care.

Several high-profile incidents illustrate the severity of these risks. At the 2013 Electronic Zoo festival in New York City, 22 attendees required emergency care and two died, primarily from MDMA-related toxicity (Ridpath et al., 2014). Similarly, the 2017 Electric Daisy Carnival (EDC) in Las Vegas documented more than 1000 medical encounters and 15 hospital transports (Note, n.d.-a; Delgado, 2017). Although national estimates specific to EDM festivals are lacking, US emergency department visits for substance-related conditions average $530 per encounter, and inpatient hospitalizations range from $9700 to $11,700 per stay, underscoring the substantial financial burden of acute drug-related morbidity (Moore et al., 2020; Note, n.d.-b).

Las Vegas hospitals, especially those located near the EDC venue, routinely experience an influx of critically ill patients during festival weekends (Note, n.d.-c; Williams, 2025). Presentations often include severe overdose, hyperthermia, dehydration, multi-organ failure, cerebrovascular ischemia, and coagulopathy (Ridpath et al., 2014). In response, festival organizers have implemented harm-reduction strategies such as shifting the event to cooler months, providing free hydration stations, shaded rest areas, and active cooling zones (Ridpath et al., 2014; Walubo and Seger, 1999). Despite these efforts, severe illness and fatalities continue to occur (Vegas, 2025).

EDM festival–related drug use poses risks not only to attendees but also to first responders and healthcare systems. Emergency departments, intensive care units, and trauma centers frequently experience operational strain during festival weekends (Eassey et al., 2024; Hospital TRM, 2025). Emergency medical services (EMS) crews experience increased call volumes, and ICUs often approach operational limits during festival weekends (Hospital TRM, 2025; Chhabra et al., 2018). These surges may delay care for non-festival patients and require resource reallocation, demonstrating the broader community-level impact of festival-related overdoses. Intensive care unit (ICU) admission is a critical indicator of overdose severity, reflecting the need for advanced airway management, hemodynamic support, and continuous monitoring. Because ICU resources are costly and limited, understanding whether festival-related overdoses disproportionately require ICU care is essential for hospital preparedness and resource planning, particularly in cities hosting recurring large-scale events (Moore et al., 2020; Note, n.d.-b; Hospital TRM, 2025).

It remains unclear whether overdoses associated with EDC are inherently more severe than those presenting to hospitals during the same time period but unrelated to the festival. Despite numerous descriptive reports documenting medical encounters at EDM festivals, few studies have systematically compared clinical outcomes, particularly ICU utilization between festival-related overdoses and non–festival-related overdoses within the same time frame and healthcare system (Ridpath et al., 2014; Chhabra et al., 2018; Black et al., 2020). Consequently, the extent to which EDC-related intoxications represent uniquely severe clinical presentations remains poorly understood. Establishing whether EDC-associated intoxications carry unique clinical risks is essential for emergency preparedness, hospital resource allocation, and targeted harm-reduction planning.

To address this gap, the present study aims to compare the clinical outcomes of patients transported from EDC with drug overdoses to those of patients presenting with non-EDC–related overdoses during the same period. By delineating the spectrum and severity of illness, this study seeks to provide evidence to inform clinical risk stratification and emergency response planning, and festival-related harm-reduction strategies.

## 2 Methods

### 2.1 Study design and setting

This retrospective cohort study was conducted at an urban tertiary care teaching hospital in Las Vegas, Nevada. The study received approval with a waiver of informed consent from the Institutional Review Board of the University of Nevada, Las Vegas (IRB No. UNLV-2024-79) and the University Medical Center of Southern Nevada (IRB No. UMC-2023-516).

#### 2.1.1 Study period and festival calendar

The analytic period spanned May 17, 2019, to May 20, 2024, encompassing all years in which the Electric Daisy Carnival (EDC) was held in Las Vegas. Because EDC was canceled in 2020 due to the Coronavirus disease 2019 (COVID-19) pandemic, no festival-related admissions occurred that year. For each EDC event, the study window included the three official festival days plus one additional day to capture delayed presentations.

### 2.2 Participants

Eligible participants were adults aged 18 years or older admitted with a primary diagnosis of drug overdose or alcohol intoxication as documented in the electronic medical record (EMR). Patients admitted under observation status, those with non–drug-related primary diagnoses, or those with insufficient documentation regarding festival attendance were excluded.

#### 2.2.1 Exposure classification

Patients were classified as either EDC-related or non-EDC–related based on EMR documentation. EDC-related cases included individuals transported directly from the festival site or those whose emergency department triage notes, emergency physician documentation, or primary inpatient team histories explicitly indicated attendance at the Electric Daisy Carnival. In routine clinical practice, festival attendance is actively elicited by triage nurses and treating physicians as part of the initial history; therefore, documentation of EDC attendance was considered reliable when present. Non-EDC cases included all other overdose admissions during the same screening windows without evidence of festival involvement.

#### 2.2.2 Clinical management

During the study period, admitted patients were managed by internal medicine and family medicine attending physicians, residents, and hospitalists. Intensive care unit (ICU) care was delivered by critical care attendings, pulmonary and critical care fellows, and internal medicine residents. Triage decisions followed standard institutional protocols. ICU admission decisions followed standard institutional criteria and were based on clinical need, including airway protection, hemodynamic instability requiring vasoactive support, severe metabolic or electrolyte abnormalities, and the need for close neurologic or cardiopulmonary monitoring.

### 2.3 Outcome measures

Primary outcomes included ICU admission and in-hospital mortality. Secondary outcomes included hospital length of stay and ICU length of stay. Clinical severity variables included Electrocardiogram (EKG) QT interval and Sequential Organ Failure Assessment (SOFA) score. ICU admission was used as a surrogate marker of critical care resource utilization.

### 2.4 Statistical analysis

Descriptive statistics were reported as means with standard deviations (M ± SD) for continuous variables and frequencies with percentages for categorical variables. Ninety-five percent confidence intervals (95% CI) were calculated where applicable. Comparisons between EDC-related and non-EDC–related overdoses were conducted using the Chi-square test for categorical variables and the independent samples *t*-test for continuous variables. Effect sizes were reported using Phi for categorical comparisons and Cohen's d for continuous variables. Logistic regression analyses were performed to identify predictors of ICU admission, with results presented as odds ratios (ORs) and 95% CIs. A *p*-value <0.05 was considered statistically significant. All statistical analyses were conducted using IBM SPSS Statistics (Statistical Package for the Social Sciences, version 29).

## 3 Results

### 3.1 Cohort and encounter characteristics

**Fig. 1:**
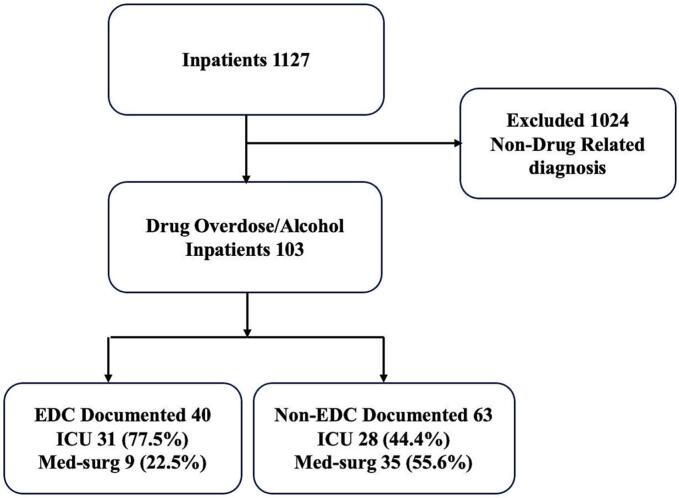
Flow Diagram of Adult Patients Presenting With Drug or Alcohol Overdose During Electric Daisy Carnival Study Period in Las Vegas, Nevada (May 2019–May 2024). During the study period, 1127 patients were admitted to the hospital. Of these, 103 met inclusion criteria for drug or alcohol overdose and comprised the final analytic cohort, including 40 EDC-related and 63 non–EDC-related cases. **Abbreviations:** ED, emergency department; EDC, Electric Daisy Carnival; ICU, intensive care unit; Med-surg, medical–surgical unit.

During the study period, 1127 patients admitted to the hospital were identified during the predefined study windows. Of these, 1024 were excluded because their primary diagnosis was non-drug-related. The remaining 103 patients met inclusion criteria and comprised the final analytic cohort. Of these, 40 (38.80%) were classified as EDC-related overdoses, and 63 (61.20%) as non-EDC–related overdoses. Among the EDC-related overdose patients, 31 (77.50%) were admitted to the ICU and 9 (22.50%) to the medical-surgical unit. Among the non-EDC–related overdose patients, 28 (44.40%) were admitted to the ICU and 35 (55.60%) to the medical-surgical unit. (Fig. 1).

The mean age was 39.70 years (SD = 12.00). Males comprised 67.00% of the cohort. BMI categories were normal weight 47.60%, overweight 34.0%, obese/morbid obese 16.50%, and underweight 1.90%. Any drug use was reported in 91.30% of patients, and multiple drug use in 44.70%. EDC-related cases accounted for 38.80% of the total sample. Mean BMI was 25.86 kg/m^2^ (SD = 4.89), mean weight 76.09 kg (SD = 17.82), mean height 171.00 cm (SD = 9.50), mean EKG QT interval 474.30 ms (SD = 46.70), and mean SOFA score 4.00 (SD = 3.30, Table 1).

**Table 1:** Values are presented as mean ± standard deviation or number (percentage)

Demographic and Clinical Characteristics of Adult Patients Hospitalized for Drug or Alcohol Overdose During the Electric Daisy Carnival Study Period in Las Vegas, Nevada (May 2019–May 2024) (*N* = 103)			
		N = 103	(95% CI)
Age (years)		39.70 ± 12.00	(37.30–42.00)
Male Sex		69 (67.00%)	(57.00–75.90)
BMI Category			
Underweight		2 (1.90%)	(0.24–6.84)
Normal		49 (47.60%)	(37.64–57.65)
Overweight		35 (34.00%)	(24.94–43.97)
Obese/Morbid Obese		17 (16.50%)	(9.92–25.11)
Any type of drug use		94 (91.30%)	(84.10–95.90)
Multiple drug use		46 (44.70%)	(34.80–54.80)
Attended EDC		40 (38.80%)	(29.40–48.90)
Weight (kg)		76.09 ± 17.82	(72.60–79.57)
Height (cm)		171.00 ± 9.50	(169.10–172.80)
BMI (kg/m^2^)		25.86 ± 4.89	(24.90–26.82)
EKG QT Interval (ms)		474.30 ± 46.70	(464.80–483.90)
SOFA Score		4.00 ± 3.30	(3.20–4.70)

**Abbreviations:** BMI, body mass index; EDC, electric daisy carnival; EKG, electrocardiogram; SOFA, sequential organ failure assessment.

EDC-related overdose patients were significantly younger than non–EDC-related overdose patients (32.40 ± 8.50 vs 44.30 ± 11.60 years, *p* < 0.001), whereas no significant differences were observed between EDC-related and non-EDC groups for sex (*p* = 0.61). Weight and BMI distribution differed significantly between groups (p < 0.001). Drug use (*p* = 0.28) and multiple drug use (*p* = 0.39) did not differ significantly. No significant differences were observed in height (*p* = 0.52), EKG QT interval (*p* = 0.72), or SOFA score (*p* = 0.47, Table 2). (See Table 3, Table 4.)

**Table 2:** Values are presented as mean ± standard deviation or number (percentage).

Comparison of Clinical and Anthropometric Characteristics Between Electric Daisy Carnival–Related and Non–Festival-Related Overdose Patients Hospitalized in Las Vegas, Nevada (May 2019–May 2024) (N = 103).					
		EDC (*N* = 40)	non-EDC (*N* = 63)	p-value	t or χ^2^ value
Age (years)		32.40 ± 8.50	44.30 ± 11.60	< 0.001	−5.57
Male Sex	Male	28 (70.00%)	41 (65.10%)	0.61	0.27
BMI Category					
	Underweight	1 (2.50%)	1 (1.60%)	< 0.001	14.86
	Normal	28 (70.00%)	21 (33.30%)		
	Overweight	9 (22.50%)	26 (41.30%)		
	Obese/Morbid Obese	2 (5.00%)	15 (23.80%)		
Any type of drug use		35 (87.50%)	59 (93.70%)	0.28	1.16
Multiple drug use		20 (50.00%)	26 (41.30%)	0.39	0.75
Weight (kg)		69.20 ± 13.60	80.44 ± 18.89	<0.001	3.25
Height (cm)		171.70 ± 9.60	170.50 ± 9.40	0.52	0.65
BMI (kg/m^2^)		23.20 ± 3.31	27.48 ± 5.06	<0.01	4.60
EKG QT Interval (ms)		476.50 ± 49.50	472.90 ± 45.10	0.72	0.36
SOFA Score		3.70 ± 2.70	4.20 ± 3.70	0.47	0.24

Abbreviations: BMI, body mass index; EDC, Electric Daisy Carnival; EKG, electrocardiogram; SOFA, Sequential Organ Failure Assessment.

**Table 3:** Values are presented as mean ± standard deviation or number (percentage).

Clinical Outcomes Among Electric Daisy Carnival–Related and Non–Festival-Related Overdose Patients Hospitalized in Las Vegas, Nevada (May 2019–May 2024) (*N* = 103).				
	**EDC (N = 40)**	**non-EDC (N = 63)**	**p-value**	**t or χ**^**2**^**value**
ICU admission, n (%)	31 (77.50%)	28 (44.40%)	< 0.001	10.93
Hospital length of stay (days)	7.00 ± 12.20	4.20 ± 3.40	0.09	1.74
ICU length of stay (days)	4.80 ± 7.30	3.60 ± 4.90	0.50	0.68
Mortality, n (%)	2 (5.00%)	2 (3.20%)	0.64	0.22

Abbreviations: ICU, intensive care unit.

**Table 4:** Reference categories were: female sex, non-EDC status.

Multivariable Logistic Regression Predicting ICU Admission Among Adult Overdose Patients Hospitalized in Las Vegas, Nevada (May 2019–May 2024) (N = 103).			
Variable	Odds Ratio	p-value	(95% CI)
Age	0.83	0.04	(0.70–0.98)
Sex	0.47	0.14	(0.17–1.29)
EDC	4.63	0.01	(1.54–13.89)
BMI (kg/m^2^)	1.51	0.05	(1.00–2.27)
Prolonged EKG QT Interval	0.99	0.68	(0.98–1.01)

Abbreviations: ICU, intensive care unit.

ICU admission differed significantly between groups (77.50% vs. 44.40%, *p* < 0.001, Phi = −0.33). No significant differences were found for total hospital length of stay (*p* = 0.09), ICU length of stay (*p* = 0.50), or mortality (*p* = 0.64).

In multivariable logistic regression analysis, age was significantly associated with ICU admission (OR = 0.83, 95% CI: 0.70–0.98, *p* = 0.04), indicating that each one-year increase in age was associated with a 17% decrease in the odds of ICU admission. EDC was also a significant predictor (OR = 4.63, 95% CI: 1.54–13.89, *p* = 0.01), with patients from EDC having more than four times the odds of ICU admission compared to those without EDC. In contrast, sex (OR = 0.47, 95% CI: 0.17–1.29, *p* = 0.14) and prolonged EKG QT interval (OR = 0.99, 95% CI: 0.98–1.01, *p* = 0.68) were not significantly associated with ICU admission after adjustment for covariates. BMI demonstrated a borderline association (OR = 1.51, 95% CI: 1.00–2.27, *p* = 0.05).

## 4 Discussion

Substance use and drug-related emergencies during large music festivals have been increasingly recognized as a significant public health challenge. Prior studies conducted at events such as Coachella in California and Tomorrowland in Belgium have consistently demonstrated high rates of presentations related to MDMA, cocaine, opioids, ketamine, and polysubstance intoxication, often complicated by hyperthermia and dehydration (Ridpath et al., 2014; Chhabra et al., 2018; Black et al., 2020). Many of these presentations are complicated by multi-organ dysfunction, underscoring the unique risks associated with high-energy outdoor settings (Palamar et al., 2019).

Our institution, located adjacent to the Electric Daisy Carnival (EDC) in Las Vegas, provides a unique vantage point for evaluating this issue. EDC is one of the largest electronic dance music festivals in the world, drawing more than 400,000 attendees annually. Local emergency medical services, hospitals, and intensive care units anticipate a surge in patients during the festival weekend (Chhabra et al., 2018). While the association between EDM festivals and drug intoxication is not new, our study is among the first to quantify hospital outcomes, specifically ICU utilization and hospital length of stay, for patients transported from EDC compared with other overdose patients.

The primary novel finding in our study is that EDC-related patients had a demonstrated higher rate of ICU admission compared with patients presenting with overdose outside of the festival context. Hospital length of stay was not statistically different but the trend toward longer stays suggests greater illness severity.

In this multivariable analysis, EDC attendance emerged as an independent predictor of ICU admission, even after adjustment for age, sex, BMI, and QT interval, suggesting that festival-associated overdoses represent a distinct clinical context associated with higher ICU utilization rather than merely reflecting demographic differences. This finding aligns with existing literature on electronic dance music festivals, which consistently reports increased rates of polysubstance use, stimulant toxicity (particularly MDMA and synthetic analogues), dehydration, and unpredictable drug adulteration, all of which may heighten clinical instability and monitoring needs. Notably, younger age was associated with increased odds of ICU admission, reinforcing prior evidence that younger festival attendees often engage in higher-intensity recreational drug use patterns rather than reflecting baseline physiologic vulnerability. The absence of significant associations with QT interval and other measured clinical parameters suggests that traditional physiologic markers may not fully capture toxicologic severity in mass-gathering settings, where uncertainty regarding substance composition and delayed toxicity may appropriately lower the threshold for ICU triage. Importantly, despite higher ICU utilization among EDC patients, mortality did not differ significantly, indicating that the impact may be driven more by resource intensity than by worse ultimate outcomes. Collectively, these findings support the growing literature emphasizing the disproportionate acute care burden imposed by large-scale events and the need for improved toxicologic surveillance during such gatherings.

Few studies have specifically examined ICU admission rates in this population. A retrospective review from New York City reported severe illness and several deaths during an EDM festival (Ridpath et al., 2014). Similarly, Australian data demonstrated toxicologically confirmed severe overdoses requiring ICU care (Black et al., 2020). These reports align with our findings and highlight that environmental stressors, drug adulteration, and polysubstance use can drive outcomes irrespective of baseline demographics. Several plausible mechanisms may explain the disproportionate ICU utilization among EDC-related patients: First-time or inexperienced use: many attendees are young adults, experimenting with illicit substances for the first time (Palamar et al., 2019). Environmental stressors: Nevada desert climate, overnight dancing, and dehydration amplify toxicity (Chhabra et al., 2018; Black et al., 2020). Polysubstance use and drug adulteration: synthetic cathinones and fentanyl analogues unpredictably increase toxicity (Black et al., 2020; Turris and Lund, 2017). Sleep deprivation and circadian disruption: overnight festival structure exacerbates risk.

From a clinical standpoint, recognition that EDC-related patients are more likely to require ICU care is highly relevant. History taking should specifically include recent festival attendance, as this may alter risk stratification. Awareness of the potential for severe hyperthermia, rhabdomyolysis, and cardiac dysrhythmias even in initially stable patients should prompt early ICU triage (Palamar et al., 2019; Ridpath et al., 2014; Black et al., 2020).

The disproportionate ICU utilization has broader public health implications. ICU admission is resource-intensive, with daily costs exceeding thousands of dollars. During EDC weekends, the increased burden can displace or delay care for non-festival emergencies such as myocardial infarction and stroke (Chhabra et al., 2018). Harm reduction strategies—hydration, cooling stations, naloxone distribution, on-site medical tents—have been recommended (Ridpath et al., 2014; Black et al., 2020) and warrant expansion in collaboration with festival organizers and local authorities.

It is also important to acknowledge system-level factors. On-site triage may filter out less severe cases, resulting in selection bias where only critically ill patients are transferred (Chhabra et al., 2018). Local practice patterns may also favor early ICU admission in anticipation of deterioration. Moreover, many attendees travel from outside the region, limiting opportunities for safe outpatient follow-up and lowering the threshold for ICU admission.

This study has several important limitations. First, its retrospective, single-center design may limit generalizability, as hospital practices, triage patterns, and local infrastructure may differ in other settings. Toxicology testing was often incomplete, and routine panels do not detect many emerging or synthetic substances, restricting our ability to fully characterize drug exposures. Additionally, several potentially important clinical and psychosocial confounders, such as psychiatric comorbidity, prior substance use history, or hydration status were not available in the medical record. The absence of a comprehensive denominator also precludes direct comparison of EDC-related overdose incidence with baseline community overdose rates. Misclassification bias is another potential concern: classification of “EDC-related” cases relied on patient disclosure and documentation, and some individuals may not have reported festival attendance. Such nondifferential misclassification would likely bias findings toward the null, meaning the observed difference in ICU utilization may underestimate the true association between festival attendance and illness severity. Finally, the modest sample size may reduce statistical power to detect smaller differences in clinical outcomes or to identify predictors in multivariable models. Despite these limitations, the study offers meaningful insights into the clinical and public health burden associated with large-scale music festivals.

Future research should validate these findings through multicenter collaborations, prospective registries, and standardized toxicology screening (Turris and Lund, 2017). Comparative studies across different festivals and climates could clarify environmental contributions. Economic analyses quantifying direct and indirect costs would strengthen the argument for preventive strategies.

In summary, our study found higher rates of ICU admission among patients transported from EDC compared with non-EDC overdose patients, and this association remained significant after adjustment for demographic and clinical covariates. These findings underscore the importance of heightened clinical vigilance, contextual awareness, and harm reduction strategies during large-scale music festivals. (Ridpath et al., 2014; Chhabra et al., 2018; Black et al., 2020).

## 5 Conclusion

These findings highlight the operational and public health impact of large-scale music festivals on local healthcare systems. Strengthening harm-reduction strategies, improving on-site medical management, and enhancing coordination between festival organizers, public health agencies, and hospitals are essential to mitigate preventable morbidity and optimize resource allocation. As Las Vegas continues to host high-volume entertainment events, these results provide important evidence to inform preparedness planning, clinical protocols, and regional initiatives.

## Declaration of generative AI and AI-assisted technologies in the manuscript preparation process

During the preparation of this work the author(s) used ChatGPT (OpenAI) in order to assist English language editing and grammatical refinement. After using this tool/service, the author(s) reviewed and edited the content as needed and take(s) full responsibility for the content of the published article.

## CRediT authorship contribution statement

**Jie Ren:** Writing – original draft, Investigation, Data curation. **Kavita Batra:** Writing – review & editing, Writing – original draft, Visualization, Validation, Supervision, Software, Project administration, Methodology, Investigation, Formal analysis, Data curation, Conceptualization. **Vidhani Goel:** Software, Formal analysis, Data curation. **Taya Jensen:** Writing – original draft, Resources. **San Tran:** Data curation. **Mutsumi Kioka:** Writing – review & editing, Writing – original draft, Visualization, Validation, Supervision, Resources, Project administration, Methodology, Investigation, Data curation, Conceptualization.

## Declaration of competing interest

The authors declare that they have no known competing financial interests or personal relationships that could have appeared to influence the work reported in this paper.

## Data Availability

Data will be made available on request.

## References

[bb0005] Alo C., Kioka M.J. (2024). Rave gone wrong: MDMA- induced medical emergency at electrical daisy carnival. A case report. Toxicol. Rep..

[bb0010] Apruzzese J.B.P., Tompkins T. (2023). Impact and hope for the live music industry. Ethnomusicology Rev..

[bb0015] Black E., Govindasamy L., Auld R., McArdle K., Sharpe C., Dawson A. (2020). Toxicological analysis of serious drug-related harm among electronic dance music festival attendees in New South Wales, Australia: A consecutive case series. Drug Alcohol Depend..

[bb0020] Chhabra N., Gimbar R.P., Walla L.M., Thompson T.M. (2018). Emergency department patient burden from an electronic dance music festival. J. Emerg. Med..

[bb0025] Eassey C., Hughes C.E., Wadds P., de Andrade D., Barratt M.J. (2024). A systematic review of interventions that impact alcohol and other drug-related harms in licensed entertainment settings and outdoor music festivals. Harm Reduct. J..

[bb0075] Delgado I. EDC medical calls up nearly 77 percent over last year. Las Vegas Review-Journal. June 19, 2017. Available from: https://www.reviewjournal.com.

[bb0030] Hospital TRM (2025).

[bb0035] JamBase (2025).

[bb0040] Moore B.J., Elixhauser A., Andrews R. (2020).

[bb0045] N. H. EDC Las Vegas 2024: 525,000 Attendees…. Digital Music News;May 23,2024

[bb0050] National inpatient hospital costs: The most expensive conditions by payer, 2017. Agency for Healthcare Research and Quality (AHRQ). 2019;HCUP Statistical Brief #261

[bb0055] Staff LVS. Las Vegas police investigating 2 deaths connected to EDC weekend. 2025;May 20

[bb0065] Palamar J.J., Acosta P., Le A., Cleland C.M., Nelson L.S. (2019). Adverse drug-related effects among electronic dance music party attendees. Int. J. Drug Policy.

[bb0070] Palamar J.J., Keyes K.M. (2020). Trends in drug use among electronic dance music party attendees in new York City, 2016-2019. Drug Alcohol Depend..

[bb0080] Ridpath A., Driver C.R., Nolan M.L., Karpati A., Kass D., Paone D. (2014). Illnesses and deaths among persons attending an electronic dance-music festival - new York City, 2013. MMWR Morb. Mortal. Wkly Rep..

[bb0085] Ridpath A.D.C., Nolan M.L. (2014). Llnesses and deaths among persons attending an electronic dance-music festival — new York City, 2013. MMWR Morb. Mortal. Wkly. Rep..

[bb0090] Turris S.A., Lund A. (2017). Mortality at music festivals: academic and Grey literature for case finding. Prehosp. Disaster Med..

[bb0095] Vegas E.L. (2025).

[bb0100] Walubo A., Seger D. (1999). Fatal multi-organ failure after suicidal overdose with MDMA, ‘ecstasy’: case report and review of the literature. Hum. Exp. Toxicol..

[bb0105] Williams A. (2025).

